# The δ-phase of SrTeO_3_ at 780 K^1^

**DOI:** 10.1107/S1600536808020151

**Published:** 2008-07-31

**Authors:** Valery E. Zavodnik, Sergey A. Ivanov, Adam I. Stash

**Affiliations:** a Karpov Institute of Physical Chemistry, 10 Vorontsovo Pole, 105064 Moscow, Russian Federation; b Materials Chemistry, Uppsala University, Box 538, SE-75121, Uppsala, Sweden

## Abstract

As part of a structural investigation of strontium tellurate(IV) (STO), SrTeO_3_, with particular emphasis on the crystal chemistry and phase transitions, the structure of the δ-phase has been determined at 780 K using a single-crystal analysis. Both structural and non-linear optical measurements indicate that STO undergoes a γ→δ second-order ferroelectric phase transition at 633 K from the *C*2 (γ) to the *C*2/*m* (δ) modification. Systematic differences between the similar γ- and δ-phase structures were determined and it was found that this phase transformation can be described by a displacive mechanism.

## Related literature

Single crystals of strontium tellurate(IV) (STO) were prepared by Sadovskaya (1984 ▶). Structural phase transitions of STO have been studied by X-ray powder diffraction by Ismailzade *et al.* (1979 ▶) and Simon *et al.* (1979 ▶), neutron powder diffraction studies have been conducted by Dityatiev *et al.* (2006 ▶) and second harmonic generation studies by Libertz & Sadovskaya (1980 ▶). The temperature dependence of the physical properties of STO was analysed by Yamada & Iwasaki (1972 ▶, 1973 ▶), Yamada (1975 ▶) and Kudzin *et al.* (1988 ▶). For related literature, see: Antonenko *et al.* (1982 ▶); Avramenko *et al.* (1984 ▶); Kudzin *et al.* (1982 ▶); Zavodnik *et al.* (2007**a* ▶,b* ▶,*c* ▶).

## Experimental

### 

#### Crystal data

SrTeO_3_
*M* *_r_* = 263.22
Monoclinic, 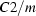

*a* = 28.438 (6) Å
*b* = 5.950 (1) Å
*c* = 15.550 (3) Å
β = 122.45 (3)°
*V* = 2220.3 (8) Å^3^
*Z* = 24
Mo *K*α radiation
μ = 22.11 mm^−1^
*T* = 780 (2) K
0.13 × 0.10 × 0.04 mm

#### Data collection

Enraf–Nonius CAD-4 diffractometer
Absorption correction: analytical (Alcock, 1970 ▶) *T*_min_ = 0.169, *T*_max_ = 0.475
1681 measured reflections
1611 independent reflections
538 reflections with *I* > 2σ(*I*)
*R*_int_ = 0.062
θ_max_ = 22.5°
3 standard reflections frequency: 60 min intensity decay: none

#### Refinement

*R*[*F* ^2^ > 2σ(*F* ^2^)] = 0.036
*wR*(*F* ^2^) = 0.095
*S* = 0.78
1611 reflections
118 parameters
Δρ_max_ = 1.22 e Å^−3^
Δρ_min_ = −1.12 e Å^−3^

### 

Data collection: *CAD-4-PC* (Enraf–Nonius, 1993 ▶); cell refinement: *CAD-4-PC*; data reduction: *CAD-4-PC*; program(s) used to solve structure: *SHELXS97* (Sheldrick, 2008 ▶); program(s) used to refine structure: *SHELXL97* (Sheldrick, 2008 ▶); molecular graphics: *DIAMOND* (Brandenburg, 2005 ▶); software used to prepare material for publication: *CIFTAB97* (Sheldrick, 2008 ▶) and *SHELXL97*.

## Supplementary Material

Crystal structure: contains datablocks I, global. DOI: 10.1107/S1600536808020151/br2075sup1.cif

Structure factors: contains datablocks I. DOI: 10.1107/S1600536808020151/br2075Isup2.hkl

Additional supplementary materials:  crystallographic information; 3D view; checkCIF report

## Figures and Tables

**Fig. 1.**
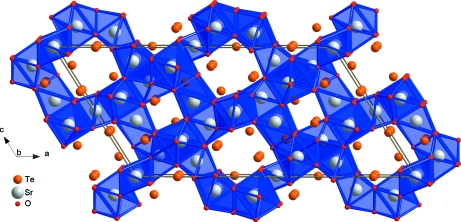
The crystal structure of δ-SrTeO3 at 780 K. The sequence of Sr polyhedra are presented, Te cations occupy two different kinds of voids in a three-dimensional lattice.

## supplementary crystallographic information

### Comment

The origin of unusual ferroelectric properties and several phase transitions between α–β (350 K), β–γ (590 K) and γ–δ (760 K) polymorphs of STO were hotly debated long time (Yamada & Iwasaki, 1973; Yamada, 1975; Simon *et al.*, 1979; Ismailzade *et al.*, 1979; Libertz & Sadovskaya, 1980, Antonenko *et al.*, 1982; Kudzin *et al.*, 1988) but the detailed structure data are lacking. Recently, in our several papers of the present series (Zavodnik *et al.*, 2007*a*,*b*,*c*,) the structures of α (T < 363 K), β (363 K < T < 563 K) and γ (563 K < T <633 K) phases STO were reported. The purpose of the present communication is to report on the structure of δ-phase and clarify the nature of γ–δ ferroelectric phase transition at 633 K. There is a number of experimental studies of dielectric, elastic, piezoelectric and optical properties near well known reversible γ–δ phase transition at 633 K (Ismailzade *et al.*, 1979; Libertz & Sadovskaya, 1980; Kudzin *et al.*, 1982, 1988; Sadovskaya, 1984; Antonenko *et al.*, 1982). All the measured constants exhibit significant changes but the lack of thermal hysteresis or phase coexistence at this transition is indicative of a second order transformation. Around 633 K the SHG signal vanishes indicating that the δ-structure is centrosymmetrical. No success was obtained in earlier attempts to determine the δ phase structure using X-ray and neutron powder diffraction studies (Simon *et al.*, 1979; Ismailzade *et al.*, 1979; Dityatiev *et al.*, 2006). The structure of δ-phase STO forms a three-dimensional lattice consisting types of irregular n-vertex SrOn (n = 6, 7, 8) polyhedra sharing corners or faces and TeO_3_ pyramidal units which share edges with Sr-polyhedra but are not connected to each other. The projection along the *b* axis (Fig. 1) shows two sorts of tunnels running along that direction. Te^4+^ cations are located inside the tunnels of different sizes and shapes which represent the required space for the lone-electron pairs within the structure. From a comparison of atomic coordinates of comparable atoms in γ and δ phases the atomic polar displacements required to achieve centrosymmetry were determined. The structures of these polymorphs are similar and the phase transformation can be realised by the orientation and tilts of the TeO_3_ pyramids and also by the variation in n-vertex SrO_n_ polyhedra without a serious changing the building structural blocks. The Te6—O31 bond length is located at distance greater than 2.8 Å and does not contribute to the first coordination sphere of Te^4+^. Probably, γ–δ phase transition in STO can be described by displacive mechanism rather than by order–disorder model. The structure–property correlation in STO is in progress and will be reported later.

### Experimental

The single crystals of STO were grown by Czochralski technique as described earlier (Libertz & Sadovskaya, 1980; Avramenko *et al.*, 1984). The products were characterized in a scanning electron microscope (Jeol 820) with an energy-dispersive spectrometer (LINK AN10000), confirming the presence and stoichiometry of Sr and Te. SHG measurements showed that there is a symmetry centre in δ-phase (which is stable above 633 K) in a full agreement with the results (Libertz & Sadovskaya, 1980).

### Refinement

The atomic coordinates of all Sr and Te cations in γ-phase were used as starting parameters. The O atoms were localized by difference Fourier maps. The selection of space group C2/m for description of crystal structure of δ-phase STO was based on the experimental data of second harmonic generation (SHG) obtained on tested single crystals. The temperature dependence of SHG signal confirms that the structure of δ-phase STO is centrosymmetric. Precise X-ray diffraction study of single crystals at high temperatures is a challenging task because there is usually only a small number of measured X-ray reflections in the data and they cover a rather limited range of sinθ/λ. At 780 K it was impossible to registrate any reflections with sinθ/λ > 0.54. The thermal vibration parameters for oxygen anions were very high and strongly anisotropic. It was difficult to use an anisotropic approximation in this high-temperature refinement because the ratio of statistically reliable reflections to a number of refined parameters was very far from an optimal value. The positive definite refinements with anisotropic atomic displacement parameters were impossible for O atoms at 780 K. It was a main reason why the oxygen atoms were refined isotropically. A special attention must be given to the accuracy of interatomic distances of Te—O which are not rather similar as in the case of α, β and γ-phases (Zavodnik *et al.*, 2007*a*,*b*,*c*). But all these Te—O bond lengths can be found acceptable if we take into account the standard deviation. The highest residual electron density peak is located 0.87 Å from atom Sr1 and the deepest hole is located 0.12 Å from atom Te4. Several atoms (Sr6, O12, O22 and O52) have increased isotropic atomic displacement parameters. These atoms are located inside significant voids which are larger than the voids for the rest of the atoms. The same peculiarity was also observed for the α- β and γ-STO structures.

### Crystal data

**Table:**

SrTeO_3_	*F*_000_ = 2736
*M**_r_* = 263.22	*D*_x_ = 4.725 Mg m^−^^3^
Monoclinic, *C*2/*m*	Mo *K*α radiation λ = 0.71073 Å
Hall symbol: -C 2y	Cell parameters from 24 reflections
*a* = 28.438 (6) Å	θ = 12.3–13.8º
*b* = 5.950 (1) Å	µ = 22.11 mm^−^^1^
*c* = 15.550 (3) Å	*T* = 780 (2) K
β = 122.45 (3)º	Triangular prism, colourless
*V* = 2220.3 (8) Å^3^	0.13 × 0.10 × 0.04 mm
*Z* = 24	

### Data collection

**Table:**

Enraf–Nonius CAD-4 with high-temperature device diffractometer	*R*_int_ = 0.062
Radiation source: fine-focus sealed tube	θ_max_ = 22.5º
Monochromator: β-filter	θ_min_ = 1.6º
*T* = 780(2) K	*h* = −30→25
ω/2θ scans	*k* = 0→6
Absorption correction: analytical(Alcock, 1970)	*l* = 0→16
*T*_min_ = 0.169, *T*_max_ = 0.475	3 standard reflections
1681 measured reflections	every 60 min
1611 independent reflections	intensity decay: none
538 reflections with *I* > 2σ(*I*)	

### Refinement

**Table:**

Refinement on *F*^2^	Secondary atom site location: difference Fourier map
Least-squares matrix: full	*w* = 1/[σ^2^(*F*_o_^2^) + (0.0453*P*)^2^] where *P* = (*F*_o_^2^ + 2*F*_c_^2^)/3
*R*[*F*^2^ > 2σ(*F*^2^)] = 0.036	(Δ/σ)_max_ = 0.001
*wR*(*F*^2^) = 0.095	Δρ_max_ = 1.22 e Å^−^^3^
*S* = 0.78	Δρ_min_ = −1.12 e Å^−^^3^
1611 reflections	Extinction correction: SHELXL97 (Sheldrick, 2008), Fc^*^=kFc[1+0.001xFc^2^λ^3^/sin(2θ)]^-1/4^
118 parameters	Extinction coefficient: 0.00009 (2)
Primary atom site location: isomorphous structure methods	

### Special details

**Table:**

Geometry. All e.s.d.'s (except the e.s.d. in the dihedral angle between two l.s. planes) are estimated using the full covariance matrix. The cell e.s.d.'s are taken into account individually in the estimation of e.s.d.'s in distances, angles and torsion angles; correlations between e.s.d.'s in cell parameters are only used when they are defined by crystal symmetry. An approximate (isotropic) treatment of cell e.s.d.'s is used for estimating e.s.d.'s involving l.s. planes.
Refinement. Refinement of *F*^2^ against ALL reflections. The weighted *R*-factor *wR* and goodness of fit *S* are based on *F*^2^, conventional *R*-factors *R* are based on *F*, with *F* set to zero for negative *F*^2^. The threshold expression of *F*^2^ > σ(*F*^2^) is used only for calculating *R*-factors(gt) *etc*. and is not relevant to the choice of reflections for refinement. *R*-factors based on *F*^2^ are statistically about twice as large as those based on *F*, and *R*- factors based on ALL data will be even larger.

### Fractional atomic coordinates and isotropic or equivalent isotropic displacement parameters (Å^2^)

**Table:**

	*x*	*y*	*z*	*U*_iso_*/*U*_eq_	
Te1	−0.01963 (9)	0.5000	0.1468 (2)	0.0530 (8)	
Te2	0.49464 (8)	0.5000	0.65585 (18)	0.0423 (7)	
Te3	0.11483 (9)	0.0000	0.2806 (2)	0.0396 (6)	
Te4	0.35697 (9)	0.0000	0.2329 (2)	0.0566 (8)	
Te5	0.14971 (9)	1.0000	−0.00115 (19)	0.0548 (8)	
Te6	0.26235 (9)	0.5000	0.41676 (19)	0.0345 (7)	
Sr1	0.12254 (12)	0.5000	0.4219 (3)	0.0458 (10)	
Sr2	0.24767 (12)	0.5000	0.1092 (3)	0.0437 (10)	
Sr3	0.24223 (13)	0.0000	0.2740 (3)	0.0507 (11)	
Sr4	0.37724 (11)	0.5000	0.3964 (3)	0.0479 (11)	
Sr5	0.12358 (13)	0.5000	0.1520 (3)	0.0501 (11)	
Sr6	0.0000	0.0000	0.0000	0.070 (2)	
Sr7	0.5000	1.0000	0.5000	0.0588 (18)	
O11	0.0554 (10)	0.5000	0.223 (2)	0.097 (9)*	
O12	−0.0384 (15)	0.317 (7)	0.050 (3)	0.277 (19)*	
O21	0.4492 (5)	0.724 (3)	0.5733 (11)	0.071 (5)*	
O22	0.5456 (15)	0.5000	0.629 (3)	0.158 (14)*	
O31	0.1641 (7)	0.231 (4)	0.3268 (14)	0.100 (6)*	
O32	0.0981 (11)	0.0000	0.148 (2)	0.100 (9)*	
O41	0.3148 (5)	−0.225 (3)	0.2374 (10)	0.071 (5)*	
O42	0.4029 (12)	0.0000	0.373 (2)	0.112 (9)*	
O51	0.1756 (6)	0.761 (3)	0.0923 (11)	0.078 (5)*	
O52	0.2055 (17)	1.0000	−0.017 (4)	0.197 (18)*	
O61	0.2317 (7)	0.5000	0.2770 (15)	0.056 (6)*	
O62	0.3118 (5)	0.737 (3)	0.4416 (11)	0.066 (5)*	

### Atomic displacement parameters (Å^2^)

**Table:**

	*U*^11^	*U*^22^	*U*^33^	*U*^12^	*U*^13^	*U*^23^
Te1	0.0488 (12)	0.0602 (19)	0.0407 (15)	0.000	0.0179 (12)	0.000
Te2	0.0397 (13)	0.0482 (17)	0.0380 (14)	0.000	0.0202 (11)	0.000
Te3	0.0456 (12)	0.0372 (15)	0.0406 (14)	0.000	0.0262 (11)	0.000
Te4	0.0529 (14)	0.0416 (18)	0.084 (2)	0.000	0.0420 (14)	0.000
Te5	0.0457 (13)	0.0585 (19)	0.0394 (14)	0.000	0.0090 (12)	0.000
Te6	0.0346 (11)	0.0316 (15)	0.0364 (14)	0.000	0.0184 (10)	0.000
Sr1	0.0464 (18)	0.030 (2)	0.045 (2)	0.000	0.0134 (16)	0.000
Sr2	0.0412 (18)	0.037 (2)	0.044 (2)	0.000	0.0167 (17)	0.000
Sr3	0.057 (2)	0.047 (3)	0.039 (2)	0.000	0.0199 (18)	0.000
Sr4	0.0411 (18)	0.049 (3)	0.051 (2)	0.000	0.0233 (18)	0.000
Sr5	0.0465 (18)	0.055 (3)	0.046 (2)	0.000	0.0234 (17)	0.000
Sr6	0.052 (3)	0.071 (5)	0.054 (4)	0.000	0.007 (3)	0.000
Sr7	0.032 (2)	0.079 (5)	0.056 (4)	0.000	0.018 (2)	0.000

### Geometric parameters (Å, °)

**Table:**

Te1—O12	1.70 (5)	Sr3—O62^iv^	2.759 (16)
Te1—O11	1.80 (2)	Sr3—O41	2.763 (16)
Te2—O22	1.71 (4)	Sr3—O51^iv^	2.801 (16)
Te2—O21	1.820 (16)	Sr3—O61	2.993 (2)
Te3—O31	1.82 (2)	Sr3—O31	3.08 (2)
Te3—O32	1.85 (3)	Sr4—O22^v^	2.43 (4)
Te4—O41	1.822 (17)	Sr4—O41^iii^	2.690 (16)
Te4—O42	1.84 (3)	Sr4—O62	2.712 (15)
Te5—O52	1.73 (4)	Sr4—O21	2.736 (15)
Te5—O51	1.879 (18)	Sr4—O42	3.132 (10)
Te6—O61	1.86 (2)	Sr5—O61	2.612 (18)
Te6—O62	1.880 (16)	Sr5—O51	2.635 (18)
Sr1—O62^i^	2.487 (15)	Sr5—O11	2.69 (3)
Sr1—O11	2.62 (3)	Sr5—O31	2.811 (19)
Sr1—O21^i^	2.651 (17)	Sr5—O12^vi^	2.95 (4)
Sr1—O31	2.83 (2)	Sr5—O32	3.054 (6)
Sr2—O52^ii^	2.42 (5)	Sr6—O32	2.49 (3)
Sr2—O51	2.469 (17)	Sr6—O12	2.50 (5)
Sr2—O41^iii^	2.493 (16)	Sr7—O42^vii^	2.38 (3)
Sr2—O61	2.88 (2)	Sr7—O21	2.802 (17)
			
O12—Te1—O12^viii^	79 (3)	O41^iii^—Sr4—O62	110.4 (5)
O12—Te1—O11	106.2 (14)	O41^vii^—Sr4—O62	73.4 (5)
O22—Te2—O21	101.7 (10)	O62^viii^—Sr4—O62	62.7 (8)
O21^viii^—Te2—O21	94.1 (10)	O22^v^—Sr4—O21	84.9 (8)
O31—Te3—O31^iii^	98.7 (12)	O41^iii^—Sr4—O21	171.5 (5)
O31—Te3—O32	97.1 (8)	O41^vii^—Sr4—O21	113.3 (6)
O41—Te4—O41^iii^	94.4 (11)	O62^viii^—Sr4—O21	103.9 (5)
O41—Te4—O42	91.1 (8)	O62—Sr4—O21	74.5 (4)
O52—Te5—O51	95.8 (11)	O22^v^—Sr4—O21^viii^	84.9 (8)
O51^ix^—Te5—O51	98.4 (10)	O41^iii^—Sr4—O21^viii^	113.3 (6)
O61—Te6—O62	94.0 (6)	O41^vii^—Sr4—O21^viii^	171.5 (5)
O62—Te6—O62^viii^	97.3 (9)	O62^viii^—Sr4—O21^viii^	74.5 (4)
O62^x^—Sr1—O62^i^	77.9 (8)	O62—Sr4—O21^viii^	103.9 (5)
O62^i^—Sr1—O11	138.1 (5)	O21—Sr4—O21^viii^	58.3 (7)
O62^x^—Sr1—O21^i^	127.0 (5)	O22^v^—Sr4—O42^vii^	72.1 (5)
O11—Sr1—O21^i^	87.1 (6)	O41^iii^—Sr4—O42^vii^	123.4 (7)
O62^x^—Sr1—O21^x^	79.8 (5)	O41^vii^—Sr4—O42^vii^	52.7 (6)
O21^i^—Sr1—O21^x^	76.6 (7)	O62^viii^—Sr4—O42^vii^	139.5 (6)
O62^x^—Sr1—O31^viii^	76.0 (5)	O62—Sr4—O42^vii^	76.8 (7)
O62^i^—Sr1—O31^viii^	117.9 (5)	O21—Sr4—O42^vii^	63.9 (7)
O11—Sr1—O31^viii^	68.1 (6)	O21^viii^—Sr4—O42^vii^	119.0 (7)
O21^i^—Sr1—O31^viii^	155.2 (5)	O22^v^—Sr4—O42	72.1 (5)
O21^x^—Sr1—O31^viii^	102.0 (5)	O41^iii^—Sr4—O42	52.7 (6)
O62^x^—Sr1—O31	117.9 (5)	O41^vii^—Sr4—O42	123.4 (7)
O62^i^—Sr1—O31	76.0 (5)	O62^viii^—Sr4—O42	76.8 (7)
O11—Sr1—O31	68.1 (6)	O62—Sr4—O42	139.5 (6)
O21^i^—Sr1—O31	102.0 (5)	O21—Sr4—O42	119.0 (7)
O21^x^—Sr1—O31	155.2 (5)	O21^viii^—Sr4—O42	63.9 (7)
O31^viii^—Sr1—O31	68.7 (8)	O42^vii^—Sr4—O42	143.6 (11)
O52^ii^—Sr2—O51	128.6 (7)	O61—Sr5—O51	66.6 (5)
O51^viii^—Sr2—O51	77.9 (8)	O51—Sr5—O51^viii^	72.2 (8)
O52^ii^—Sr2—O41^vii^	92.5 (8)	O61—Sr5—O11	120.9 (7)
O51^viii^—Sr2—O41^vii^	137.2 (5)	O51—Sr5—O11	143.9 (4)
O51—Sr2—O41^vii^	84.7 (6)	O61—Sr5—O31^viii^	64.7 (5)
O41^vii^—Sr2—O41^iii^	82.1 (8)	O51—Sr5—O31^viii^	89.4 (6)
O52^ii^—Sr2—O61	160.0 (10)	O51^viii^—Sr5—O31^viii^	131.2 (5)
O51—Sr2—O61	64.6 (5)	O11—Sr5—O31^viii^	67.5 (6)
O41^vii^—Sr2—O61	72.6 (4)	O61—Sr5—O31	64.7 (5)
O62^viii^—Sr3—O62^iv^	69.0 (7)	O51—Sr5—O31	131.2 (5)
O62^viii^—Sr3—O41^iii^	71.6 (4)	O51^viii^—Sr5—O31	89.4 (6)
O62^iv^—Sr3—O41^iii^	103.4 (4)	O11—Sr5—O31	67.5 (6)
O41^iii^—Sr3—O41	57.9 (7)	O31^viii^—Sr5—O31	69.3 (9)
O62^viii^—Sr3—O51^iv^	173.7 (5)	O61—Sr5—O12^vi^	139.2 (9)
O62^iv^—Sr3—O51^iv^	114.8 (6)	O51—Sr5—O12^vi^	98.0 (10)
O41^iii^—Sr3—O51^iv^	102.3 (5)	O51^viii^—Sr5—O12^vi^	72.8 (9)
O41—Sr3—O51^iv^	73.9 (5)	O11—Sr5—O12^vi^	94.2 (9)
O62^viii^—Sr3—O51^viii^	114.8 (6)	O31^viii^—Sr5—O12^vi^	155.8 (9)
O62^iv^—Sr3—O51^viii^	173.7 (5)	O31—Sr5—O12^vi^	119.6 (10)
O41^iii^—Sr3—O51^viii^	73.9 (5)	O61—Sr5—O12^xi^	139.2 (9)
O41—Sr3—O51^viii^	102.3 (5)	O51—Sr5—O12^xi^	72.8 (9)
O51^iv^—Sr3—O51^viii^	61.0 (8)	O51^viii^—Sr5—O12^xi^	98.0 (10)
O62^viii^—Sr3—O61^iv^	125.1 (5)	O11—Sr5—O12^xi^	94.2 (9)
O62^iv^—Sr3—O61^iv^	56.6 (5)	O31^viii^—Sr5—O12^xi^	119.6 (10)
O41^iii^—Sr3—O61^iv^	125.2 (6)	O31—Sr5—O12^xi^	155.8 (9)
O41—Sr3—O61^iv^	67.3 (5)	O12^vi^—Sr5—O12^xi^	43.2 (17)
O51^iv^—Sr3—O61^iv^	59.5 (5)	O61—Sr5—O32	100.8 (5)
O51^viii^—Sr3—O61^iv^	120.1 (5)	O51—Sr5—O32	137.6 (7)
O62^viii^—Sr3—O61	56.6 (5)	O51^viii^—Sr5—O32	65.8 (6)
O62^iv^—Sr3—O61	125.1 (5)	O11—Sr5—O32	78.1 (5)
O41^iii^—Sr3—O61	67.3 (5)	O31^viii^—Sr5—O32	122.9 (7)
O41—Sr3—O61	125.2 (6)	O31—Sr5—O32	55.7 (7)
O51^iv^—Sr3—O61	120.1 (5)	O12^vi^—Sr5—O32	64.5 (10)
O51^viii^—Sr3—O61	59.5 (5)	O12^xi^—Sr5—O32	106.6 (11)
O61^iv^—Sr3—O61	167.5 (8)	O61—Sr5—O32^vii^	100.8 (5)
O62^viii^—Sr3—O31	75.4 (4)	O51—Sr5—O32^vii^	65.8 (6)
O62^iv^—Sr3—O31	104.8 (5)	O51^viii^—Sr5—O32^vii^	137.6 (7)
O41^iii^—Sr3—O31	124.4 (6)	O11—Sr5—O32^vii^	78.1 (5)
O41—Sr3—O31	176.4 (5)	O31^viii^—Sr5—O32^vii^	55.7 (7)
O51^iv^—Sr3—O31	107.6 (5)	O31—Sr5—O32^vii^	122.9 (7)
O51^viii^—Sr3—O31	81.2 (5)	O12^vi^—Sr5—O32^vii^	106.6 (10)
O61^iv^—Sr3—O31	110.4 (6)	O12^xi^—Sr5—O32^vii^	64.5 (10)
O61—Sr3—O31	57.2 (5)	O32—Sr5—O32^vii^	153.8 (10)
O62^viii^—Sr3—O31^iii^	104.8 (5)	O32—Sr6—O32^xii^	180.0 (11)
O62^iv^—Sr3—O31^iii^	75.4 (4)	O32—Sr6—O12^xii^	80.0 (9)
O41^iii^—Sr3—O31^iii^	176.4 (5)	O32—Sr6—O12^iii^	100.0 (9)
O41—Sr3—O31^iii^	124.4 (6)	O32^xii^—Sr6—O12^iii^	80.0 (9)
O51^iv^—Sr3—O31^iii^	81.2 (5)	O12^xii^—Sr6—O12^iii^	82 (2)
O51^viii^—Sr3—O31^iii^	107.6 (5)	O12^xii^—Sr6—O12^vi^	98 (2)
O61^iv^—Sr3—O31^iii^	57.2 (5)	O12^iii^—Sr6—O12^vi^	180 (2)
O61—Sr3—O31^iii^	110.4 (6)	O32—Sr6—O12	100.0 (9)
O31—Sr3—O31^iii^	53.2 (8)	O42^v^—Sr7—O42^vii^	180.0 (11)
O22^v^—Sr4—O41^iii^	93.3 (8)	O42^v^—Sr7—O21^xiii^	73.6 (7)
O41^iii^—Sr4—O41^vii^	75.0 (7)	O42^vii^—Sr7—O21^xiii^	106.4 (7)
O22^v^—Sr4—O62^viii^	148.0 (4)	O21^xiii^—Sr7—O21^xiv^	71.8 (7)
O41^iii^—Sr4—O62^viii^	73.4 (5)	O21^xiv^—Sr7—O21	108.2 (7)
O41^vii^—Sr4—O62^viii^	110.4 (5)	O21^xiv^—Sr7—O21^ix^	180.0 (5)
O22^v^—Sr4—O62	148.0 (4)		

Symmetry codes: (i) −*x*+1/2, *y*−1/2, −*z*+1; (ii) −*x*+1/2, −*y*+3/2, −*z*; (iii) *x*, −*y*, *z*; (iv) *x*, *y*−1, *z*; (v) −*x*+1, −*y*+1, −*z*+1; (vi) −*x*, *y*, −*z*; (vii) *x*, *y*+1, *z*; (viii) *x*, −*y*+1, *z*; (ix) *x*, −*y*+2, *z*; (x) −*x*+1/2, −*y*+3/2, −*z*+1; (xi) −*x*, −*y*+1, −*z*; (xii) −*x*, −*y*, −*z*; (xiii) −*x*+1, −*y*+2, −*z*+1; (xiv) −*x*+1, *y*, −*z*+1.
